# Validity of geographic-level social determinant of health metrics in pancreatic neuroendocrine tumors

**DOI:** 10.1530/EO-25-0029

**Published:** 2025-05-15

**Authors:** Andrea Gillis, Brendon Herring, Rachael Guenter, Weisheng Chen, Dai Chen, Herbert Chen, John Bart Rose, Upender Manne, Smita Bhatia

**Affiliations:** ^1^ University of Alabama at Birmingham, Department of Surgery, Birmingham, USA; ^2^ University of Alabama at Birmingham, Department of Pediatrics, Birmingham, USA; ^3^ University of Alabama at Birmingham, Department of Pathology, Birmingham, USA

**Keywords:** pancreatic neuroendocrine tumors, social determinants of health, environmental exposure, health outcome disparities

## Abstract

Various social determinants of health (SDOH) metrics, also known as area-based social measures, are utilized to evaluate access to cancer care and to explain disparities in outcomes. Little prior work has compared the validity of these various geographic metrics. We reviewed all patients surgically treated for PNETs (2006–2022) at a single comprehensive cancer center. We collected patient demographics including self-reported race (White or Black), billing addresses, tumor characteristics and area-based social measures. We then compared between- and within-race differences to understand accuracy across different geographic levels. One hundred seventy-nine patients were included; 49 (27%) Black, a median age of 60.3 years and 86 (48%) females. At the block group/census tract level, compared to White patients, Black patients lived in neighborhoods with lower educational attainment, lower income, higher rates of uninsurance, higher overall social vulnerability index (SVI), and higher area deprivation index (ADI) (all *P* < 0.05). These differences, however, were masked when examining county-level area-based social measures. Compared to census block group/tract-level data, for White patients, zip code-level metrics underestimated income and overestimated uninsurance level (*P* < 0.05). County-level metrics underestimated White patients’ income and education level but overestimated poverty, uninsurance rate and SVI (all *P* < 0.05). For Black patients, zip code-level metrics overestimated poverty and uninsurance rates (*P* < 0.05); the only inaccurate county-level metric was overestimation of SVI (*P* < 0.001). Black patients with PNETs experience more vulnerable area-based social measures, a disparity which may be hidden when analyzing large geographic metrics.

## Introduction

Pancreatic neuroendocrine tumors (PNETs) are the second most prevalent pancreatic malignancy, with an estimated 4,032 new cases diagnosed annually and rising (Yao *et al.* 2008, Lubner *et al.* 2011, Ren *et al.* 2019, Wang *et al.* 2019, Gosain *et al.* 2020, Wu *et al.* 2024). Unfortunately, disparities exist in PNET treatment and outcomes, with Black patients suffering worse outcomes than their White peers (DePalo *et al.* 2019, Xu *et al.* 2021, Zheng-Pywell *et al.* 2022, Holland & Rose 2024, Mirza *et al.* 2024). The explanation for this is likely multifactorial and may include differences in access to care and tumor biology (Herring *et al.* 2022).

One way to explore the causes (and potential solutions) of disparities between demographic groups is by analysis of exposure to adverse social determinants of health (SDOH). These are area-based environmental and societal factors that influence patient outcomes and are known to disproportionately affect racial minorities (Notterman & Mitchell 2015). They may be collected at a variety of geographic levels (Notterman & Mitchell 2015, Schillinger 2020, Johnson *et al.* 2021). There is evidence that area-based social measures such as rural residence and single marital status are associated with delayed presentation and poorer PNET outcomes, but this has not been explored as a driving factor for the observed racial disparities in outcome (Cheung 2013, Gosain *et al.* 2020).

In order to answer this question, one must examine the potential mediating effects of adverse area-based social measures on racial disparities in outcomes. Area-based social measures are often measured using the United States Census in order from smallest to largest geographic region: individual reporting, census block group, census tract, zip code and county (Diez-Roux *et al.* 2001, Krieger *et al.* 2002, Fontenot *et al.* 2018). Large national or state-level registries (i.e., SEER and NCDB) provide area-based social measures primarily at larger geographic area levels (i.e., zip code, county) in order to simplify analyses, maximize anonymity and limit the labor-intensive process of gathering individual-level data. This lack of granularity limits applicability of results to individual patients. There are no generally agreed upon validated measures of the most influential area-based social measures on outcome, and this likely varies by population of study and disease state.

We often assume that individual-level measures will correlate with area-based social measures (Diez-Roux *et al.* 2001). However, this can lead to dangerous overgeneralizations especially when comparing races (Shavers 2007, Pearson *et al.* 2023). Little prior work has been done in the general or a cancer population to confirm this or compare the various area-based social measures by geographic area. PNET in particular is a malignancy that affects populations uniquely, given it has no screening criteria and is usually found incidentally (Harrelson *et al.* 2023). This begs the question of whether previously published conclusions using large-scale registries for patients with PNET are valid or generalizable for different races (Fouad *et al.* 2017, Xu *et al.* 2021).

The objective of this study was to compare the accuracy and validity of area-based social measures for Black and White patients utilizing single-institutional data in a racially diverse PNET population. To understand and quantify differences in area-based social measures, we compared between- race differences across different geographic levels. We also compared these area based social measures within races to understand the accuracy of each metric when compared to the gold standard of individual, census block group and tract-level metrics. We hypothesized that smaller geographic levels would be most accurate for both races, with a stepwise decrease in accuracy among larger-level measures. Understanding the characteristics of different area-based social measures will enable future discernment when evaluating their impact on driving racial disparities in outcomes for patients with PNET and other cancer patient populations.

## Materials and methods

This study was approved by the University of Alabama at Birmingham Institutional Review Board: #300001622. We completed a retrospective review of all patients surgically treated for sporadic PNETs from 2006 (start of electronic medical record system) to 2022 at a single NCI-designated cancer center in the Southern United States. We collected patient demographics including self-reported race (White or Black; other groups excluded due to low numbers), age, sex, insurance, billing address at time of surgery, distance from treating facility, tumor characteristics (size, grade, surgical margins, functional status, lymph node involvement and presence of metastasis) and area level social measures. White and Black races were studied because they were the largest racial groups treated at our center and nationally. In addition, these groups have the most well- described outcome disparities. We excluded neuroendocrine carcinomas and high-grade (G3) tumors as these aggressive tumors are less representative of the overall PNET population.

Billing addresses were geocoded and matched to area-level social metrics including census block group, census tract, county-level or five-digit zip code-level data collected from the year closest to their date of surgery. Census block groups are defined by the US Census as small collections of ‘blocks’ or neighborhoods spanning the entire United States (https://www.census.gov/programs-surveys/geography/about/glossary.html). Block groups consist of about 60–3,000 individuals and are the smallest geographic census unit that provides publicly available area-based social measures. Census tracts are larger statistical subdivisions of a county defined by population density. With population sizes between approximately 1,200 and 8,000 people, they are relatively constant and only updated at the time of every decennial census. Here, census block group and census tract levels are combined and described as ‘neighborhood level’ measures.

Neighborhood-level area-based social measures served as the gold standard. Individuals served as their own controls by comparing their neighborhood-level data to their data from the county and zip code levels. Then, data were compared at every geographic level between White and Black patients. Individual social metrics included marital status and insurance type.

SDOH metrics were selected using the Office of Disease Prevention and Health Promotion ‘Healthy People 2030’ domain framework (Office of Disease Prevention and Health Promotion 2025) (Table 1) including: i) education access and quality (percent of geographic level with high school (HS) diploma by age 25), ii) health care access and quality (insurance rate of community and individual), iii) neighborhood and built environment (ADI), iv) social and community context (SVI), and v) economic stability (community-level income and percent of area living below the federal poverty level) (Office of Disease Prevention and Health Promotion 2025). ADI is a composite metric published by the University of Wisconsin and organized by census block group or zip code level (Center for Health Disparities Research 2021). ADI is generated from 17 publicly available environmental and demographic data points and reported as a composite percentile score from 1 (least socioeconomically disadvantaged) to 100 (most disadvantaged). The most recent version was released in 2021.

**Table 1:** Detailed description of SDOH domains measured.

Domain measured	Metric and source	Year available	Geographic unit
Economic stability	Median household income (ACS)	2015, 2018, 2020	County, zip code, block group
	Poverty level (% living below the federal poverty level, ACS)	2015, 2018, 2020	County, zip code, census tract
Education access and quality	Education attainment (% graduated HS or equivalent among persons ≥25 years, ACS)	2015, 2018, 2020	County, zip code, block group
Healthcare access and quality	Small area health insurance estimates (% of adults <65 without health insurance, ACS), insurance provider	2015, 2018, 2020	Individual, county, zip code, census tract
Neighborhood/built environment	ADI	2015, 2021	Census block group ONLY
Social/community context	SVI, age, sex, race	2010, 2012, 2014, 2016, 2018, 2020	Individual, census tract or county level

ACS, American Community Survey; HS, high school; ADI, area deprivation index; SVI, social vulnerability index.

The SVI was created by the Centers for Disease Control and Prevention (CDC) & the Agency for Toxic Substances and Disease Registry (ATSDR) and uses 16 US census variables to identify communities vulnerable to the negative effects of human-caused or natural disasters (Centers for Disease Control and Prevention 2024). It has been reported at the US Census Tract or County level every 2 years since 2010. Higher SVI percentiles indicate more vulnerability. Both ADI and SVI have been investigated as SDOH metrics for disparities in cancer care (Centers for Disease Control and Prevention 2024, Center for Health Disparities Research 2021, Mora *et al.* 2021, Singh 2003, Tran *et al.* 2023).

### Statistical analysis

Descriptive statistics were completed for all patients, including cohort means, medians and standard deviation/interquartile range as appropriate. Normality was assessed with histograms. Data that were normally distributed were described using parametric tests such as *t*-tests and chi-squared tests. Non-normally distributed data were analyzed using Wilcoxon rank sum tests. Data were analyzed using R and SAS 9.4 software and a two-sided *P* value of <0.05 was considered significant.

Power analysis was completed for each of the SDOH domains (Dziak *et al.* 2020). For median household income at the block level: with 130 White patients and a median of $58.1 K (standard error of $23.3 K), and 49 African American patients with a median neighborhood income of $40.1 K (standard error of $21.4 K), we yielded a >90% power to show the difference between the two race groups with a significant level of 0.05. The calculation for the other domains was similar: a power of 88.5% to show the difference in neighborhood poverty between groups, a power of 79.6% to show the difference in neighborhood HS graduation rate, and a power of 81.3% to show a difference in neighborhood uninsurance rates (*P* < 0.05).

## Results

### Overall cohort demographics

A total of 179 patients were surgically treated for PNET at our institution, including 49 (27%) patients who self-identified as Black (Table 2). The median age at surgery was 60 (IQR: 51–68) years and 86 (48%) patients were female. Fifty percent of patients had private insurance coverage. At the neighborhood level, the overall cohort’s median HS graduation rate was 87.4% (IQR: 78.9–94.0%), median population living below the federal poverty level was 15.3% (IQR: 9.8–21.4%), median income was $49 K (IQR: $35.3–66.3 K), median SVI was the 50th percentile (IQR: 30th–70th percentile) and median ADI was the 62nd percentile (IQR: 43rd–83rd percentile).

**Table 2:** Baseline pancreatic neuroendocrine tumor patient demographics by race.

Demographic	Overall (*n* = 179)	African American/Black (*n* = 49)	White (*n* = 130)	*P*-value
Age at time of surgery, median (IQR)	60.3 (50.9–67.7)	57.2 (47.8–64.6)	62.3 (52.9–68.6)	**0.009**
Gender				
Female	93 (52.0%)	34 (69.4%)	59 (45.4%)	**0.004**
Male	86 (48.0%)	15 (30.6%)	71 (54.6%)	
BMI (mean +/−SD)	31.4 +/−8.2	31.7 +/−9.7	31.3 +/−7.7	0.812
Married	126 (70.4%)	25 (51.0%)	101 (77.7%)	**0.004**
Insurance type				
Private	93 (50.0%)	25 (48.1%)	68 (50.7%)	0.953
Governmental	84 (45.2%)	23 (44.2%)	61 (45.5%)	
Uninsured	4 (2.2%)	1 (1.9%)	3 (2.2%)	
Distance to treating facility, median (miles, IQR)	78.5 (36.5–120.6)	88.9 (54.2–139.3)	73.9 (33.2–102.7)	0.078
Tumor size (cm, mean ± SD)	3.4 ± 2.8	4.1 ± 3.7	3.1 ± 2.4	**0.031**
Surgical margin status: involved	27 (15.3%)	8 (16.7%)	19 (14.7%)	0.9441
Differentiation: well	126 (71.2%)	33 (68.8%)	93 (72.1%)	0.840
Tumor grade: 1	80 (45.2%)	20 (41.7%)	60 (46.5%)	0.8122
Tumor grade: 2	57 (32.2%)	15 (31.3%)	42 (32.6%)	
Tumor grade: unknown	40 (22.7%)	13 (27.6%)	27 (20.9%)	
Functional PNET	24 (13.4%)	5 (10.2%)	19 (14.6%)	0.270
Metastatic disease	21 (11.7%)	7 (14.3%)	14 (10.8%)	0.332

Bold indicates statistical significance, *P* < 0.05.

Black patients were younger at diagnosis (median 57 years) compared to White patients (median 62 years, *P* = 0.009) and were more likely to be female (69 versus 45%, *P* < 0.01). Tumor sizes among Black patients were larger, with a mean tumor size of 4.1 cm compared to 3.1 cm (*P* = 0.03) among White patients.

### Between race neighborhood level differences

Compared to White patients, Black patients with PNETs more frequently lived in disadvantaged neighborhoods with lower educational attainment (median HS graduation 84 versus 88%), lower income (median household $34 K versus $53 K), higher poverty rate (median 21 versus 14%), higher uninsured rate (20 versus 15%) and higher overall SVI (60th vs 50th percentile) (all *P* < 0.05) (Table 3). State-level ADI was significantly higher among Black patients (60th percentile, IQR: 30–90) compared to White patients (30th percentile, IQR: 20–60) (*P* < 0.001).

**Table 3:** Differences in area-based social measures by race (*n* = 179).

	Neighborhood			Zip code			County		
	AA/Black	White	*P*-value	AA/Black	White	*P*-value	AA/Black	White	*P*-value
Median household income (in $1,000s) (IQR)	$33.7 ($25.8–$51.2)	$52.8 ($41.0–$71.1)	**<0.01**	$34.4 ($30.3–$45.8)	$47.3 ($37.5–$61.6)	**<0.01**	$45.0 ($37.7–$45.8)	$45.6 ($39.2–$51.3)	0.44
Median % HS graduate (IQR)	84.1 (75.5–92.6)	87.9 (80.2–95.0)	**0.02**	82.4 (76.5–88.1)	85.1 (79.8–91.1)	**0.03**	86.6 (79.3–88.4)	85.4 (79.3–88.4)	0.43
Median % impoverished (IQR)	21.4 (13.8–30.3)	13.7 (7.6–18.8)	**<0.01**	25.2 (18.9–32.6)	15.2 (9.8–20.7)	**<0.01**	18.8 (17.4–23)	18.8 (15.6–21.3)	0.59
Median % uninsured (IQR)	20.2 (13.2–24.3)	15.2 (11.4–21.4)	**0.04**	22.1 (17.8–25.2)	17.2 (13.4–20.4)	**<0.01**	17.1 (15.5–20.7)	18.6 (17.1–21.5)	0.09
Median SVI percentile (IQR)	0.6 (0.5–0.9)	0.5 (0.2–0.6)	**<0.01**				0.3 (0.1–0.5)	0.3 (0.1–0.5)	0.08
Median national ADI	80 (60–93)	55 (39–77)	**<0.01**						

AA, African American; ADI, area deprivation index; SVI, social vulnerability index. Block group-level measures: household income and graduation rate. Census tract-level measures: SVI, % uninsured, % impoverished. Bold indicates statistical significance, *P* < 0.05.

These differences, however, were masked when comparing county-level social measures. At the county level, there appeared to be no significant differences in the number of patients from marginalized social groups by race for educational attainment, income, insurance rate or SVI (all *P* > 0.05).

At the zip code level, there were significant differences in the number of patients from marginalized social groups between races for all metrics (*P* < 0.05). However, differences were exaggerated between races for poverty and insurance status, with seemingly larger differences between groups than when compared to the differences at the neighborhood level (*P* < 0.05) (Table 3).

### Within race neighborhood level differences

When examining within-group differences among White patients, compared to the neighborhood level, the zip code-level metrics underestimated neighborhood income and insurance rates (*P* < 0.05) (Table 4). County-level metrics underestimated White patients’ neighborhood median household income, insurance rates and HS graduation rates, but overestimated poverty rates and SVI (all *P* < 0.05). For Black patients, compared to the neighborhood level, zip code-level metrics overestimated poverty level and rates of the uninsured (*P* < 0.05). Only SVI was overestimated at the county level (*P* < 0.001).

**Table 4:** Differences in area-based social measures by geographic level (*n* = 179).

	Neighborhood level metric*	Zip code level metric*	*P*-value	Neighborhood level metric*	County evel metric*	*P*-value	Zip level	County level	*P*-value
**African American/Black**									
Median household income (in $1,000s) (IQR)	$33.7 ($25.8–$51.2)	$34.4 ($30.3–$45.8)	0.315	$33.7 ($25.8–$51.2)	$45.0 ($37.7–$45.8)	0.237	34.4 (30.3–45.8)	45.0 (37.7–45.8)	**<0.001**
Median % HS graduate (IQR)	84.1 (75.5–92.6)	82.4 (76.5–88.1)	0.736	84.1 (75.5–92.6)	86.6 (79.3–88.4)	0.150	82.4 (76.5–88.1)	86.6 (79.3–88.4)	0.055
Median % impoverished (IQR)	21.4 (13.8–30.3)	25.2 (18.9–32.6)	**0.042**	21.4 (13.8–30.3)	18.8 (17.4–23)	0.156	25.2 (18.9–32.6)	18.8 (17.4–23)	**<0.001**
Median % uninsured (IQR)	20.2 (13.2–24.3)	22.1 (17.8–25.2)	**0.043**	20.2 (13.2–24.3)	17.1 (15.5–20.7)	0.558	22.1 (17.8–25.2)	17.1 (15.5–20.7)	**<0.001**
Median SVI percentile (IQR)		NA		0.6 (0.5–0.9)	0.3 (0.1–0.5)	**<0.001**	NA		
**White**									
Median household income (in $1,000s) (IQR)	$52.8 ($41.0–$71.1)	$47.3 ($37.5–$61.6)	**<0.01**	$52.8 ($41.0–$71.1)	$45.6 ($39.2–$51.3)	**<0.001**	47.3 (37.5–61.6)	45.6 (39.2–51.3)	**<0.001**
Median % HS graduate (IQR)	87.9 (80.2–95.0)	85.1 (79.8–91.1)	0.061	87.9 (80.2–95.0)	85.4 (79.3–88.4)	**0.008**	85.1 (79.8–91.1)	85.4 (79.3–88.4)	0.103
Median % impoverished (IQR)	13.7 (7.6–18.8)	15.2 (9.8–20.7)	0.139	13.7 (7.6–18.8)	18.8 (15.6–21.3)	**<0.001**	15.2 (9.8–20.7)	18.8 (15.6–21.3)	**<0.001**
Median % uninsured (IQR)	15.2 (11.4–21.4)	17.2 (13.4–20.4)	**<0.01**	15.2 (11.4–21.4)	18.6 (17.1–21.5)	**<0.001**	17.2 (13.4–20.4)	18.6 (17.1–21.5)	**<0.01**
Median SVI percentile (IQR)				0.5 (0.2–0.6)	0.3 (0.1–0.5)	**<0.01**			

SVI, social vulnerability index. Block group-level measures: household income and graduation rate. Census tract-level measures: SVI, % uninsured, % impoverished. Bold indicates statistical significance, *P* < 0.05.

* All between-race differences *P* < 0.05.

## Discussion

Moving the needle on race- and socioeconomic status-based disparities in health outcomes is an ongoing challenge. Race-based differences in outcomes are often the easiest to capture, likely due to convenience; most EMRs and cancer registries report race (Boffa *et al.* 2017, Doll *et al.* 2018). However, race is a social construct that only tells part of the story and often a part that is not clearly actionable at the individual patient level (Schillinger 2020). Disparities outcomes research is moving toward capturing the intersection of environmental adverse determinants of health that differentially impact minority populations such as housing discrimination, racism and differential economic access (Ward *et al.* 2004). In order to study these exposures and develop interventions to address them for patients with PNET, they must be accurately and reproducibly measured (Amini *et al.* 2024, Casey *et al.* 2024). The ideal area-based social measures would be gathered at the patient level, but historically this has not been captured in large EMRs or registries. There are currently no guidelines specifying the most valid area measure. As a proxy, researchers often use zip code- or county-level area-based social measures of adversity (Dasari *et al.* 2017). However, there are little data on the validity of this and in which ways these larger area metrics are inaccurate. Our study shows that these large geographic area-based social measures may be inaccurate in unpredictable ways, especially for different races.

Our results show that, in general, large area (zip code, county) based social measures for White patients treated for PNET lead to inaccurate conclusions such as an apparent lower household income, higher level of uninsured and lower level of education when compared to neighborhood-level metrics. County-level metrics were much less accurate than zip code-level metrics. For Black patients, analyzing larger geographic units at times caused these patients to seem more vulnerable, but overall the results were more accurate than those for White patients when compared to neighborhood-level metrics.

When examining between-group differences, using larger geographic units erased the racial differences in the proportion of vulnerable populations. These results are novel and draw attention to the limited validity of large geographic units, especially when examining racial disparities. Although these large area-level metrics are more convenient to use, they may dilute or erase the clinically meaningful differences in rates of marginalized social groups by race. This may also explain why some prior PNET studies demonstrate racial and socioeconomic disparities (Gosain *et al.* 2020, Zheng-Pywell *et al.* 2021), while others do not (Marincola Smith *et al.* 2020).

This study is also unique in that it only includes a surgical cohort, which may have differential access to care compared to other epidemiological studies of cancer patients. The benefit here is that all patients have a billing address, which allows direct comparison between geographic areas. The findings of this study can inform future outcomes-based research.

In future studies, we caution cancer researchers to seek and utilize the smallest geographic unit of the most robust metric of vulnerability when studying healthcare disparities. These metrics may be used in analyses as an outcome or covariate, and researchers need to be rigorous with the application of these data. In order to reduce disparities, we have to understand what we are measuring and how accurate it may be. Otherwise, misleading, paradoxical or inaccurate conclusions may be drawn. Guidelines and researchers in the field of health outcomes should understand the limitations of large area geographic measurements of unmet needs and should design future registries and trials that collect small area or individually measured metrics; otherwise, conclusions may be unreliable and unreproducible. The NIH All of Us registry is an excellent example of this by preferentially collecting patient-reported, directly measured EMR and survey data to push forward robust health outcomes research (Denny *et al.* 2019). Although these are more resource-intensive approaches, the present study demonstrates the importance of this approach in NETs as well as other cancer populations.

There are some limitations in this study. We only included patients who underwent surgical resection for PNETs, which may not be generalizable to patients with unresectable tumors or other malignancies. This is a single-institution study, which may have limited generalizability to other centers. Our institution is also situated in the Southern United States, which is a unique region compared to the rest of the United States. This includes overrepresentation of Black and African American identifying patients (26% in Alabama) and a larger amount of persistently impoverished and rural neighborhoods compared to the rest of the United States (Savage *et al.* 2013, Davis *et al.* 2014, Gyawu *et al.* 2015). This may emphasize how large geographic area metrics are even more unreliable in diverse patient cohorts. In addition, we utilized patient billing addresses at the time of surgery, which may not be the patient’s true home address but is likely an adequate proxy (Adkins-Jackson *et al.* 2022). Finally, this is a study of an uncommon neoplasm (Fowler *et al.* 2024). However, this is a useful cohort to examine because PNET diagnosis is not linked to access to screening but overall access to care and surgery, representing a disease that is appropriate to measure baseline representation of vulnerable social groups.

## Conclusions

Compared to White patients, Black patients with PNETs are more likely to be from vulnerable social groups, a disparity that may be hidden when analyzing large geographic regional variations. For the most robust conclusions about racial disparities driving outcomes in cancer research, investigators must prioritize area-based social measures for individuals that utilize the smallest geographic region.

## Declaration of interest

The authors declare that there is no conflict of interest that could be perceived as prejudicing the impartiality of the work reported.

## Funding

This work was supported by the National Cancer Institutehttps://doi.org/10.13039/100000054 grant: 3U54CA118948-17S1.

## Author contribution statement

AG, JBR, UM and SB were responsible for project conception. AG, BH, RG and WC contributed to data collection and analysis. CD conducted the statistical analysis. HC, AG, RG, JBR and SB were involved in manuscript writing and revision.
